# Thomas James Fahy, MD, FRCPsych

**DOI:** 10.1192/bjb.2023.36

**Published:** 2024-02

**Authors:** Brendan D. Kelly

Formerly Professor of Psychiatry, University College, Galway, Ireland

**Figure:**
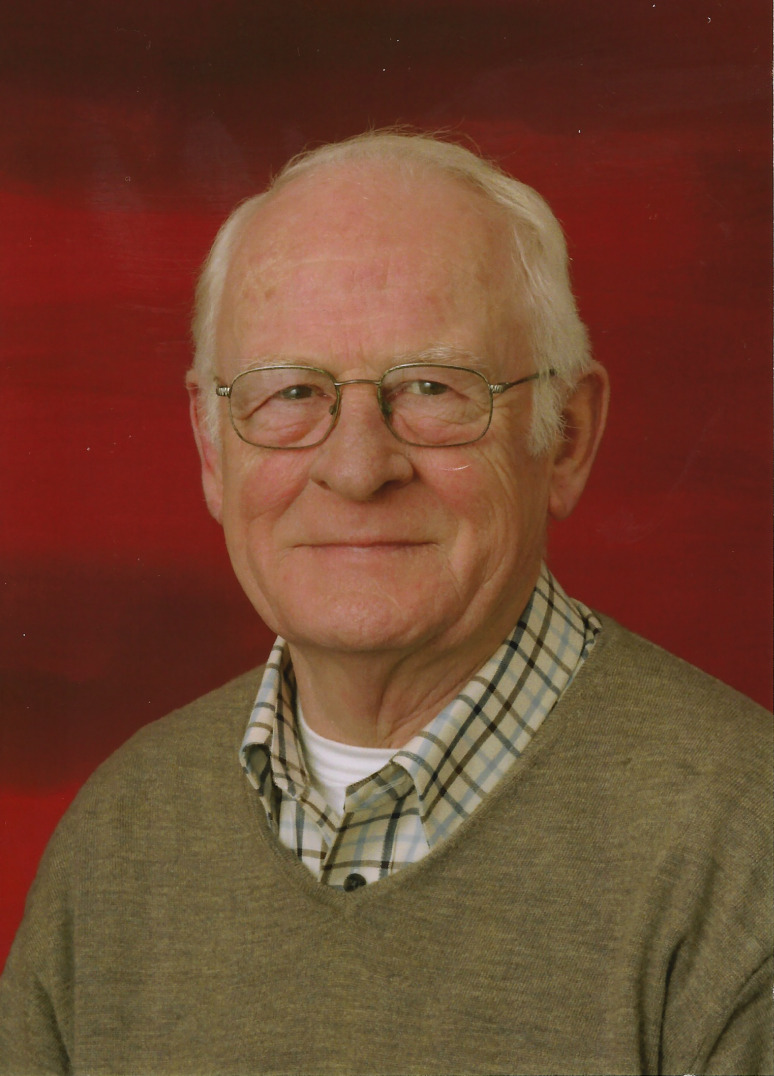

Thomas James Fahy, who died on 9 January 2023, aged 86, following a long illness, made enormous contributions to the development of psychiatry in England and Ireland. A Foundation Fellow of the Royal College of Psychiatrists, Tom was deeply involved in College activities and was Founder Chairman of the Irish Psychiatric Training Committee. He was Professor of Psychiatry at University College, Galway, from 1975 to 2001 and served on the Irish Medical Council and Medical Research Council, among other bodies.

He led the modernisation of clinical services in Galway and linked his academic work with the development of evidence-based care. In 1982, he co-authored a substantial study, *Electroconvulsive Therapy in the Republic of Ireland*, which showed that Irish psychiatrists’ attitudes towards ECT were broadly similar to those in Great Britain, although there was less emphasis on complex issues of informed and valid consent in Ireland, and premises and equipment varied considerably across the country.^1^ The report presented recommendations to the Irish Division of the Royal College of Psychiatrists relating to both training and clinical practice, with particular emphasis on informed consent.

Tom was born in The Curragh, County Kildare, to Peter, an Irish army ophthalmologist, and Kathleen, a housewife. After education in Castleknock School in Dublin, he read medicine at University College Dublin and qualified in 1959. He pursued further training in medicine and psychiatry in Birmingham with Professor Sidney Brandon and then Newcastle upon Tyne with Professor Martin Roth, where he met Ann (née Colvin), an anaesthetist, whom he married in 1967. He returned to Dublin as Clinical Director at St Loman's Hospital in 1968. He received his Doctorate in Medicine (MD) in 1969 for work on the phenomenology of depression in hospital and the community.

Tom spent a sabbatical year at White Plains Hospital, New York, in 1971, after which he was offered an Associate Professorship with a guaranteed Chair of Psychiatry at Cornell University within 2 years. Instead, he took up the Chair of Psychiatry at Galway in 1975. Over the course of his career, Tom made substantial contributions to clinical practice, undergraduate teaching, postgraduate training and academic research in the areas of head injury, anxiety disorders, post-traumatic stress disorder and suicide.

A gifted speaker, Tom brought extraordinary creative and critical rigour to academic psychiatry in Ireland and was responsible in no small part for the high academic standards in Irish psychiatry at the start of the 21st century. In September 2001, he retired as Clinical Director of the West Galway Psychiatric Service and from the Chair of Psychiatry in Galway.

He loved living in the West of Ireland with Ann and his family. He took great pleasure in a number of activities, including travelling wine regions of France, horses and the Galway Blazers, fishing rivers and Lough Corrib for salmon and trout, golf, gardening, watching rugby and reading Irish history. His family remember how grateful he was to have had the opportunity to work and live in an area he called ‘a great part of the world’.

He is survived by his wife, their daughters Kathleen, Bebe and Alice and four grandchildren, Aoife, Orla, Joseph and Lilian.
